# Introduction to the special issue “Redesigning the hypotheses for drug‐resistant epilepsy”

**DOI:** 10.1002/epi4.12582

**Published:** 2022-02-04

**Authors:** Luisa Rocha

**Affiliations:** ^1^ Pharmacobiology Department Center for Research and Advanced Studies México City México

**Keywords:** drug‐resistant seizures, epilepsy

## Abstract

Drug‐resistant epilepsy represents an important neurological condition. Its prevalence is not modified in spite of the different hypotheses established to understand the mechanisms involved. The special issue “Redesigning the hypotheses for drug‐resistant epilepsy” represents an effort of different investigators to discuss the limitations of the hypotheses that explain the drug resistance in epilepsy. In addition, new paradigms and novel strategies to control drug‐resistant epilepsy are pointed out to understand this condition.

Almost every significant breakthrough in the field of scientific endeavor is first a break with tradition, with old ways of thinking, with old paradigms. Stephen R. Covey

Drug‐resistant epilepsy affects approximately one‐third of the patients with epilepsy. Managing these patients is a challenge especially in developing countries such as those from Latin‐America, where the prevalence and incidence rates of epilepsy in the general population are higher when compared with other countries such as United States.^1^

Several experimental models have been used to understand the mechanisms involved in drug‐resistant epilepsy and to identify novel therapies to control this condition. Moreover, the mechanisms involved are explained by specific hypotheses, which are not mutually exclusive.^2^ However, the global prevalence of this disease persists without changes.

The present Special Issue of Epilepsia Open contains critical reviews focused to discuss the limitations of the different hypotheses used to explain the drug‐resistant epilepsy. For example, these hypotheses as well as the different experimental models designed for the screening of antiseizure medications do not consider factors such as age, sex, type of epilepsy, and comorbid disorders.^3^ In addition, these hypotheses do not contemplate critical conditions such as neuroinflammation that facilitates the brain excitability and cell damage. Epigenetic changes or protein‐protein interactions with alterations of signaling transduction can explain the lack of response to diverse antiseizure medications with different targets or receptors.^4^ A critical review focuses to support the expression of high‐frequency oscillations as a novel biomarker of intrinsic seizure severity in patients with drug‐resistant seizures.^5^

We discuss the relevance of considering the drug‐resistant phenotype in epilepsy as a complex and multifactorial condition that should be approached through an integrative view. It also supported the relevance of different multiomic modalities to understand the basic mechanisms involved in the drug resistance condition of patients with mesial temporal lobe epilepsy.^6^

A novel notion is that the drug resistance phenotype is associated with changes in the central and peripheral expression of multidrug transporters proteins.^7^ On the other hand, overexpression of multidrug transporter proteins can result from the administration of antiseizure medications.^8^ Indeed, it is suggested that this situation can be avoided changing the regimen of administration of the antiseizure medications or using adjuvant drugs that downregulate the expression of transporters.

Additional critical reviews focus to evaluate new paradigms and novel strategies to control drug‐resistant epilepsy. One of them presents evidence supporting nanomedicine as a promising alternative to augment the effectiveness of antiseizure medications into the brain of patients with drug‐resistant epilepsy. It also presents the state of the art of controlled drug delivery systems designed to increase drug penetration into the brain parenchyma, such as polymeric and lipid‐based nanocarriers.^9^ In addition, a critical review encourages the use of tailored multitarget drugs as well as partial or low‐affinity agonists as a novel approximation to control drug‐resistant seizures.

It is important to mention that this Supplement is the result of the efforts of Latin‐American investigators that participated in the 3th Latin‐American Workshop on Neurobiology of Epilepsy “REDISIGNING THE HYPOTHESES FOR DRUG‐RESISTANT EPILEPSY.” This workshop was carried out during the 10th Latin‐American Congress of Epilepsy. It was designed to address and discuss important concepts associated with the drug‐resistant condition with the purpose to obtain novel and low‐cost strategies to control the epilepsy according to the economical situation of the region.

Finally, I confirm that I have read the Journal's position on issues involved in ethical publication and affirm that this report is consistent with those guidelines.

## CONFLICT OF INTEREST

I have no conflicts of interest to disclose. I confirm that I have read the Journal's position regarding the ethical publication and affirm that this report is consistent with those guidelines.
